# Bcl-2 proteins, cell migration and embryonic development: lessons from zebrafish

**DOI:** 10.1038/cddis.2015.286

**Published:** 2015-10-15

**Authors:** J Prudent, N Popgeorgiev, B Bonneau, G Gillet

**Affiliations:** 1 Montreal Neurological Institute, McGill University, 3801 University Street, Montreal, Québec QC H3A 2B4, Canada; 2 Centre de recherche en cancérologie de Lyon, U1052 INSERM, UMR CNRS 5286, Centre Léon Bérard, Université Lyon I, Université de Lyon, 28 rue Laennec, Lyon 69008, France; 3 Laboratory for Developmental Neurobiology, Brain Science Institute, Riken, Wako, Saitama 351-0198, Japan

B-cell lymphoma-2 (*Bcl-2*) was cloned 30 years ago and associated with B-cell follicular lymphoma. A number of Bcl-2 homologs were identified later on. Importantly, the Bcl-2 family was found to control the mitochondrial outer membrane permeabilization: a key step of the mitochondrial pathway of apoptosis.^1^ Bcl-2 homologs are evolutionarily conserved throughout metazoans and considered as the hallmarks of multicellularity. Genetic manipulation in nematodes and mice demonstrated that the *bcl-2* family has a pivotal role in tissue homeostasis by controlling cell death; however, an increased number of *in vitro* studies have identified additional non-apoptotic functions, suggesting that Bcl-2 proteins are in fact multitask factors (Figure 1a).^2^

Besides their mitochondrial localization, Bcl-2 proteins are also found in the endoplasmic reticulum (ER). In fact, a number of them contribute to apoptosis regulation though the control of Ca^2+^ exchanges at the level of the ER/mitochondria interface. Indeed physical proximity between these organelles creates intracellular microdomains, considered as Ca^2+^ hotspots.^3^ Mitochondria constantly uptake Ca^2+^ to ensure their physiological functions; they are also able to rapidly uptake Ca^2+^ when massively released from the ER, acting as a genuine Ca^2+^ buffer. This fast accumulation may lead to mitochondrial Ca^2+^ overload and, depending on Ca^2+^ levels, the cells will undergo apoptosis or necrosis.^4^ Bcl-2 proteins control Ca^2+^ exchanges through direct interactions with ER Ca^2+^ channels and pumps including the Inositol 1,4,5-Trisphosphate receptor (IP_3_R), the Ca^2+^-ATPase (SERCA) pump, the ryanodine receptor, the Bax inhibitor-1 channel, as well as, the voltage-dependent anion channel (VDAC) at the mitochondria (reviewed in Bonneau *et al.*^2^). It was reported that overexpression of Bcl-2 may lead to a decrease of the ER Ca^2+^ load,^5^ and the ability of Bcl-2 proteins to regulate intracellular Ca^2+^ homeostasis was linked to non-apoptotic functions (Figure 1a).

## Bcl-2 proteins, Ca^2+^ and zebrafish development

**Figure 1:**
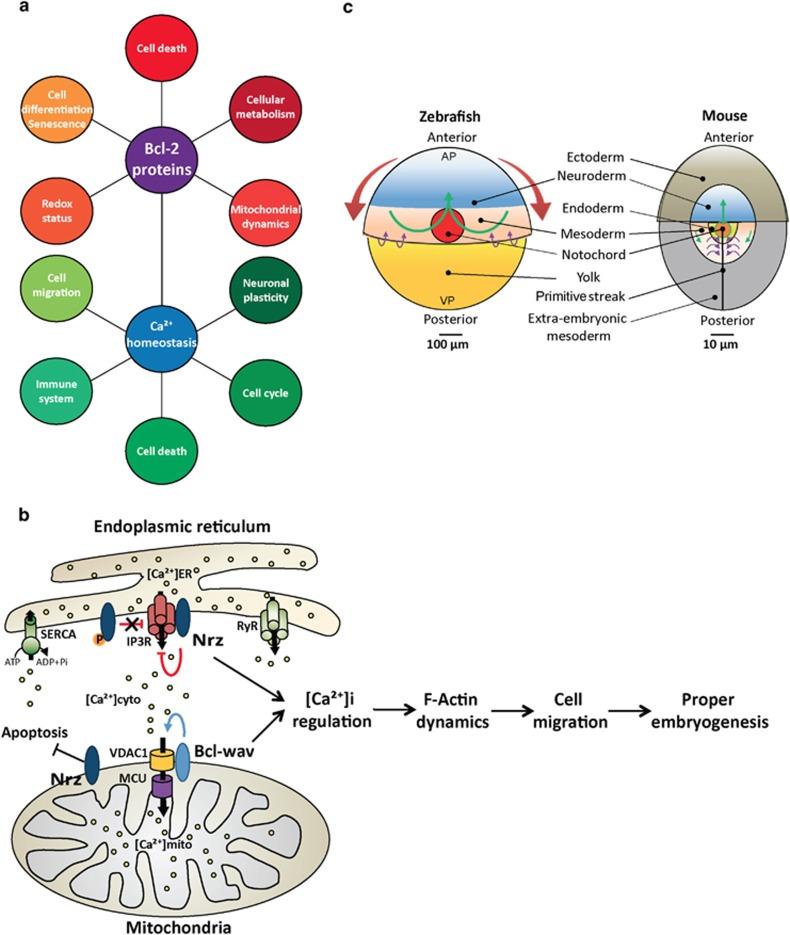
Non-apoptotic functions of the Bcl-2 family of proteins. (**a**) Simplified representation of Bcl-2 proteins functions. Bcl-2 proteins are multitask factors and linked to non-apoptotic functions. The top panel represents the different Ca^2+^-independent functions linked to the Bcl-2 family of proteins, whereas the bottom panel highlights roles that may be attributed to Bcl-2 proteins, thanks to their capacity to regulate intracellular Ca^2+^ homeostasis (reviewed in Bonneau *et al.*^2^). (**b**) Nrz and Bcl-wav regulate cell migration and zebrafish embryonic development by the control of intracellular Ca^2+^ fluxes via their interactions with IP_3_R and VDAC1, respectively. Invalidation of either *nrz* or *bclwav* leads to an increase of cytosolic Ca^2+^ level inside the embryo inducing cytoskeleton defects. These two proteins are involved in crucial morphogenetic movements, which occur during gastrulation, whereas, surprisingly, they do not appear to be involved in apoptosis control at that stage. Interaction of Nrz with IP_3_R is regulated by Nrz phosphorylation, which disrupts Nrz/IP_3_R interaction and leads to ER-Ca^2+^ release. Nrz can also be localized to mitochondria where it prevents apoptosis.^7^ This function is not described here as it is not related to cell migration and appears to be important only at later stages of zebrafish development. Only the interacting partners of the Bcl-2 family of proteins directly involved in the regulation of Ca^2+^ trafficking are represented. (**c**) Scheme of the dorsal view of zebrafish (7 h post fertilization) and mouse (6.5 days post fertilization) gastrulae. During early zebrafish development, cells migrate towards the vegetal pole in a movement called epiboly (dark red arrows). At 50% epiboly, at the dorsal side, cells start invaginating (purple arrows). Notochord precursor cells migrate actively towards the dorsal and the anterior–posterior poles in a movement called convergence-extension (CE; green arrows). In comparison, the mouse embryo does not show epiboly morphogenesis. CE movements are much smaller, compared with zebrafish. AP: animal pole; VP: vegetal pole

To further comprehend the roles of Bcl-2 proteins linked to Ca^2+^ fluxes regulation, we took advantage of the zebrafish. This model possesses all key components of the vertebrate cell death machinery and represents a suitable model for studying Ca^2+^ trafficking during embryogenesis.^6, 7^ Different studies already highlighted the role of zebrafish Bcl-2 proteins in apoptosis. However, their roles on Ca^2+^ homeostasis and their precise functions during early development are still unknown. We paid attention to two Bcl-2 homologs: Nrz (Nr-13 ortholog in zebrafish) and Bcl-wav, a new evolutionary conserved Bcl-2 homolog in bony fishes and anurans. These genes exhibit opposite effects regarding apoptosis, but both orchestrate the actin cytoskeleton dynamics via the regulation of Ca^2+^ homeostasis (Figure 1b).

Nrz is an apoptosis inhibitor which is critical for the first morphogenetic cell movements during gastrulation (epiboly). Epiboly is characterized by migration of the embryonic cells from the top of the yolk sac towards the vegetal pole of the embryo to envelop the entire yolk (Figure 1c). It is in part driven by a contractible actin–myosin ring at the leading edge of the migrating cell layer.^7^ Knockdown of *nrz* leads to embryonic death, independent of apoptosis, resulting from the detachment of the entire embryonic cells from the yolk sac. At the molecular level, Nrz directly interacts with IP_3_R1 to decrease Ca^2+^ release from the ER. In fact, *nrz* silencing leads to a massive release of Ca^2+^ in the yolk syncytial layer, which induces premature contraction of the actin–myosin ring that squeezes the embryo.^7^ Moreover, to ensure epiboly progression, Nrz/IP_3_R1 interaction is tightly regulated by Nrz phosphorylation.^8^ Indeed, Nrz phosphorylation inhibits its interaction with IP_3_R1, which contributes to the fine tuning of ER-Ca^2+^ release.

On the other hand, silencing of *bclwav*, a pro-apoptotic member, leads to marked developmental defects, including shortening and deviation of the antero–posterior axis.^6^ This phenotype is owing to the major alterations of convergence and extension (CE) movements during gastrulation, independently of cell death. Whereas during CE, embryonic stem cells migrate synchronously to establish the major embryonic axis (Figure 1c), in *bclwav*-null embryos the coordinated migration of these cells is impaired because of the alteration of the dynamics and polarity of F-actin protrusions. Interestingly, this phenotype is correlated with alterations of mitochondrial Ca^2+^ buffering. Actually, Bcl-wav was found to enhance mitochondrial Ca^2+^ uptake by direct interaction with VDAC1, thus indirectly controlling actin dynamics and cell migration.^6^

## Role of MCU in early stages of development: lessons from zebrafish model

It is important to note that *mcu* knockdown phenocopies the loss of *bclwav* in zebrafish, providing a physiological function of MCU (mitochondrial calcium uniporter) and giving insight into the role of mitochondrial Ca^2+^ oscillations in vertebrate development.^6^ Unexpectedly, *mcu* knockout in the mouse results in a rather mild phenotype with overall size reduction, decreased effort capacities and metabolic alterations, but no major developmental defects.^9^

Thus intracellular Ca^2+^ oscillations may have variable importance during embryogenesis, depending on the egg type and size. Indeed, during the development of teleosteans, including zebrafish, eggs have huge amounts of yolk stocks and undergo incomplete cleavage. The cells located at the animal pole of the embryo need to undergo a complex set of morphogenic movements including epiboly and CE (Figure 1c). During epiboly, long-range Ca^2+^ waves propagate through the entire embryonic margin, which appears to precede the actin–myosin ring formation.^10^ It is tempting to speculate that, in this case, Ca^2+^ signaling ensures long-range cell–cell communication and synchronous cell migration. In contrast, mammalian embryos have negligible yolk amounts and lack epiboly movements, Ca^2+^ signaling contributing to short-distance communications. Thus it seems that the zebrafish model is of particular interest to study the role of cell migration regulators during early development.

## Bcl-2 proteins, Ca^2+^ and mouse development

Knockouts (KO) in mice, confirmed that most of Bcl-2 proteins are primarily involved in cell death regulation. However some phenotypes cannot be explained by this sole effect. For instance *bcl-2*-KO leads to reduced size and growth retardation (reviewed in Roset *et al.*^11^), a phenotype also observed in *mcu*-KO mice. Another example is the apoptosis inhibitor *mcl-1* whose invalidation leads to a lethal peri-implantation phenotype characterized by implantation defaults of the blastocyst (reviewed in Roset *et al.*^11^). Interestingly, this process is mediated by trophoblast cells, which form cytoplasmic lamelipodia like projections that pass through the zona pellucida and interact with the external environment.^12^ Ca^2+^ signaling seems to be required for trophoblast adhesion as chelation of intracellular Ca^2+^ or inhibition of the Ca^2+^-dependent proteins significantly decreases trophoblast-binding activity. Conversely, blastocyst cavitation is accelerated by elevation of intracellular Ca^2+^ and occurred predominantly via IP_3_R.^13^ Moreover, isolated *mcl-1*-deficient blastocysts showed no increased apoptosis, suggesting that *mcl-1* might have additional uncharacterized functions. Interestingly, Mcl-1 was described to regulate intracellular Ca^2+^ homeostasis by interacting with IP_3_R.^14^ Recently, it was shown that Mcl-1 may also interact with VDAC to increase mitochondrial Ca^2+^ level and promote cell migration.^15^ This raises the possibility that a physiological function of Mcl-1 may be linked to intracellular Ca^2+^ trafficking to permit blastocyst adhesion during mouse embryonic development.

In light of the multiple functions of these proteins beyond apoptosis and results obtained with the zebrafish model it may be worthwhile to revisit the *bcl-2* KO mice phenotypes focusing on early developmental steps. Thus research performed on zebrafish confirms that Bcl-2 proteins are not only involved in cell death but may control cell migration in a Ca^2+^-dependent manner during embryonic development, contributing to extend our knowledge about the multiple roles of this fascinating family of proteins.
